# RNAi-mediated down-regulation of *SHATTERPROOF* gene in transgenic oilseed rape

**DOI:** 10.1007/s13205-014-0226-9

**Published:** 2014-05-22

**Authors:** Hadis Kord, Ali Mohammad Shakib, Mohammad Hossein Daneshvar, Pejman Azadi, Vahid Bayat, Mohsen Mashayekhi, Mahboobeh Zarea, Alireza Seifi, Mana Ahmad-Raji

**Affiliations:** 1 Department of Tissue culture and Genetic Engineering, Agricultural Biotechnology Research Institute of Iran (ABRII), Karaj, Iran; 2 Ramin University of Agricultural and Natural Resources, Ahvaz, Iran

**Keywords:** *BnSHP* gene, Gene silencing, Oilseed rape, RNAi, Pod shattering

## Abstract

Oilseed rape is one of the important oil plants. Pod shattering is one of the problems in oilseed rape production especially in regions with dry conditions. One of the important genes in Brassica pod opening is *SHATTERPROOF1* (*SHP1*). Down-regulation of *BnSHP1* expression by RNAi can increase resistance to pod shattering. A 470 bp of the *BnSHP1* cDNA sequence constructed in an RNAi-silencing vector was transferred to oilseed rape cv. SLM046. Molecular analysis of T2 transgenic plants by RT-PCR and Real-time PCR showed that expression of the *BnSHP* alleles was highly decreased in comparison with control plants. Morphologically, transgenic plants were normal and produced seeds at greenhouse conditions. At ripening, stage pods failed to shatter, and a finger pressure was needed for pod opening.

## Introduction

Oilseed rape (*Brassica napus* L.) is the third most important oilseed crop in the world (Basalma 2008). Seeds have about 40–48 % oil with a high amount oleic acid and low linolenic acid suitable for frying applications and cooking. Dehiscence of pods causes significant yield loss (Raman et al. 2011). Ordinary yield losses are in the range of 10–25 % (Price et al. 1996). Seed losses have been reported as much as 50 % of the expected yield when adverse climatic conditions delayed harvesting (Macleod 1981; Child and Evans 1989). The process of pod shatter begins with degradation and separation of cell walls along a layer of few cells, termed the dehiscence zone (Meakin and Roberts 1990). Resistance to shattering is an important and necessary trait for oilseed rape improvement (Kadkol 2009). Attempts to solve this problem by interspecific hybridization using related species such as *B. nigra*, *B. juncea* and *B. rapa* have been faced with some difficulties as other undesirable traits will be integrated too (Prakash and Chopra 1990; Kadkol 2009). In Arabidopsis, which is in the same family of brassicaceae, several genes including the ALCATRAZ (ALC), INDEHISCENT (IND), *SHATTERPROOF1* (*SHP1*) and *SHATTERPROOF2* (*SHP2*) and *FRUITFUL* (*FUL*) have been shown to be involved in pod dehiscence (Raman et al. 2011). Genes for a number of hydrolytic enzymes, such as endopolygalacturonases, have also roles in dehiscence (Petersen et al. 1996). In Arabidopsis, *SHP* genes are specifically expressed in flowers with strong expression in the outer replum (Savidge et al. 1995; Flanagan et al. 1996). *SHP* gene also has mainly effect in the ripening of strawberries (Daminato et al. 2013). In *B. napus*, three *BnSHP* alleles (*BnSHP1*, *BnSHP2a* and *BnSHP2b*) have been identified (Tan et al. 2009). *BnSHP1* and *BnSHP2* show 80 % identity at nucleotide sequence. The expression of *BnSHP2a* and *BnSHP2b* (Two alleles of *BnSHP* gene which differ only in downstream sequences) are mainly in root, floral buds and pods, and most strongly in floral buds (Tan et al. 2009). It is suggested that less severe phenotype of indehiscence will be better, and the *SHP*, *IND* and *ALC* genes are ideal candidates for research and application in breeding new lines suitable for mechanized harvest (Liljegren et al. 2000; Tan et al. 2009). Recent advances about the role of MADS-box genes in dehiscence zone development have been reviewed (Ferrándiz and Fourquin 2014). In this study, we report the effect of the silencing cassette on expression of *BnSHP* alleles in transgenic oilseed rape plants using RNAi approach.

## Materials and methods

### Nucleic acid isolation

DNA was isolated from 100 mg leaf tissues using the procedure of Dellaporta et al. (1983). For RNA isolation, total RNA was extracted from floral buds using RNeasy Mini Kit (Qiagene Co.). The quantity and quality of RNA samples were checked using nano spectrophotometry and agarose gel electrophoresis. First-strand cDNA was synthesized using 2 µg of total RNA with iScript Select cDNA synthesis kit (Bio-rad Co.) in a 20-µl reaction using oligo-dT’s according to manufacturer’s instructions.

### Construction of RNAi cassette

A 470-bp fragment of the *BnSHP1* cDNA (Accession, AY036062) without MADS-box region was amplified by PCR using specific primers; F: 5′-ATACTAGTGGCGCGCCCCGTTAACCCTCCACTG-3′ and R: 5′-GCCTTAATTAAATTTAAATTTGAAGAGGAGGTTGGTC-3′ containing restriction enzyme digestion sites for *Asc*1, *Aws*1, *Spe*1 and *Pac*1 (underlined), for cloning the sense and antisense fragments in the above sites in pGSA1252 behind the CaMV35S promoter. The RNAi cassette was removed with *Pst1* digestion and sub-cloned in the *Pst1* site in pCAMBIA3301 to make pCAMRNAi.

### Production of transgenic plants

*Agrobacterium tumefaciens* strain AGL0 containing the plasmid pCAMBIA3301 was used for transformation. Cotyledon explants of rapeseed cv. SLM046 were inoculated and co-cultivated with Agrobacterium inoculum on MS medium containing 1 mg/l 2,4-D and 4.5 mg/l BAP. After co-cultivation, cotyledonary explants were transferred to MS selection medium, containing 4.5 mg/l BAP and 4 mg/l phosphinothricine^,^ 400 mg/l cefotaxime and 300 mg/l carbeniciline. The regenerated plants were analyzed by histochemical GUS assay according to the method reported by Jefferson et al. (1987).

The rooted transgenic plants were transferred into a mixture of peat and perlite (1:1, v/v), and they were grown in the greenhouse conditions. At five-leaf stage, the plants were incubated at 4 °C for 8 weeks to vernalize, and then they were moved to 25 °C for 16 h in light and 8 h in dark till maturity. The presence of transgene in T1 transgenic plants was confirmed by amplification of *BnSHP* sense, antisense cassette (F: 5′-AATACTAGTGGCGCGCCCCGTTAACCC TCCTACTG-3′, R: 5′-GCCTTAATTAAATTTAAATTTGAAGAGGAGGTTGGTC-3′, underlined part is a tail segment) and *bar* (F: 5′-ATCTCGGTGACGGGCAGGAC-3′, R: 5′-CGCAGGACCCGCAGGAGTG-3′) by PCR. A T_2_ transgenic line (cultured seeds from T1 line) was used for gene expression evaluation.

To study the expression of *BnSHP* alleles (*SHP1, SHP2*-*a* and *SHP2*-*b*), pod samples were taken from three transgenic plants of a T_2_ line and one non-transgenic plants (two replications from each plant) for RNA extraction. RT- and Real-time PCR was conducted with two specific primer pairs:

P14 F: 5′-TGAACTAGTCCATGGAGATCTTCTTCTCATGATCAGTCGCAGCATT-3′, P14 R: 5′-AGCTTAATTAAATTTAAATTTAAACAAGTTGAAGAGGAGGTTGG-3′ (producing a 151-bp fragment from three alleles, underlined part is a tail segment); P19 F: 5′-GAACAAGGCGCGAGATTGAATCC-3′ and P19 R: 5′-GATCATGAGAAGAAGACAGACCGG-3′ (producing a 94-bp fragment from *SHP1*). The *GAPDH* gene-specific primers: F: 5′-AGAGCCGCTTCCTTCAACATCATT-3′ and R: 5′-TGGGCACACGGAAGGACATACC-3′ (producing a 112-bp fragment) were used as a reference gene. The relative gene expression data were analyzed using the 2^−∆∆CT^ method as described previously (Schmittgen and Livak 2008). The amount of *BnSHP* gene expression in transgenic and non-transgenic plants was calculated using the threshold data by 2^−∆∆CT^ method (Pfaffl et al. 2002), and the data were statistically analyzed, and the graphs were drawn by Bio-Rad software package.

## Results and discussion

### Construction of RNAi cassette and transgenic plant production

**Fig. 1:**
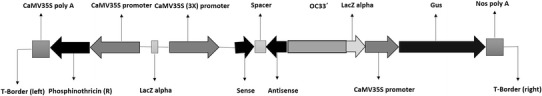
Schematic representation of the silencing cassette for the *BnSHP* gene. A 470-bp fragment of the *BnSHP1* cDNA was cloned into pGSA1252 vector as sense and antisense segments, and the cassette was sub-cloned in pCAMBIA3301 transformation vector. CaMV35S: 35S promoter of a cauliflower mosaic virus, OCS3′: octopine synthase gene, GUS: β-glucuronidase gene, NOS A: terminator of the nopaline synthase gene

**Fig. 2:**
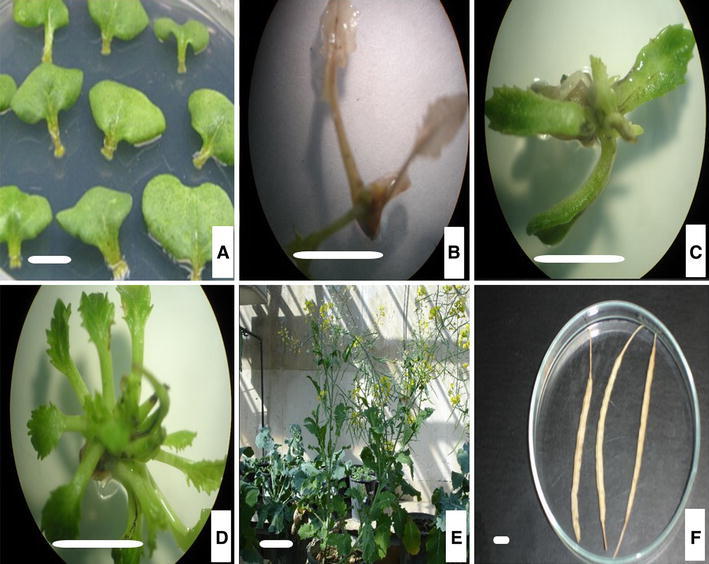
Development of transgenic *B. napus*. **a** Explants on shoot induction medium supplemented with 400 mg/l cefotaxime and 300 mg/l carbeniciline. **b** Non-transformed shoot on selection medium **c, d** Putative transgenic shoot on MS medium supplemented with 4.5 mg/l BAP, 4 mg/l phosphinothricine, 400 mg/l cefotaxime and 300 mg/l carbeniciline). **e** Flowering and fruiting (pod development) of transgenic plants in the greenhouse (*Bar*: 10 cm). **f** Ripening pods. *Bars***a**, **b**, **c**, **d** and **f**: 1 cm

**Fig. 3:**
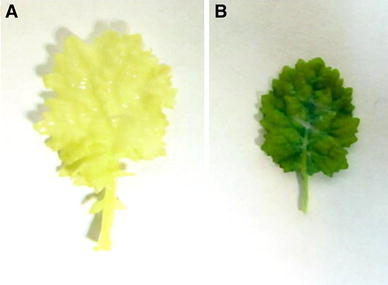
Histochemical GUS assay in transgenic plant. Leaf of non-transformed plant (**a**) and stable GUS expression (**b**) of *B. napus* transgenic plant. *Bars* 5 mm

**Fig. 4:**
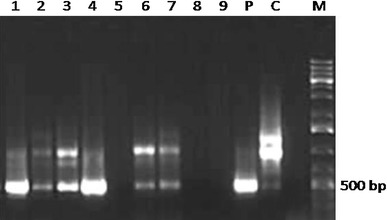
Detection of the antisense segment of *BnSHP*RNAi cassette in T0 putative transgenic *B. napus* plants. A 501-bp antisense fragment of the *BnSHP* gene was amplified using PCR. *Lanes* 1–4 and 6–7 produced clear bands of 501 bp for the transgene (higher band is related to the internal *SHP* gene); 5–8–9 blank; *P* positive control (plasmid vector of transformation); *C* control (non-transgenic plant); *M* 1 kb Plus DNA Ladder

**Fig. 5:**
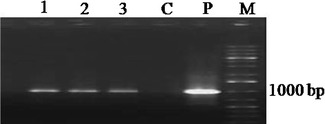
Detection of the *GUS* gene in T1 transgenic *B. napus* plants. A 1000-bp fragment of the *GUS* gene was amplified using PCR. *Lanes* 1–3 transgenic plants; *C* control (non-transgenic plant); *P* positive control (plasmid vector of transformation); *M* 1 kb Plus DNA Ladder

To construct the RNAi cassette, a 506-bp fragment (containing a 470-bp sequence downstream the MADS-box region of the *BnSHP1* cDNA) was amplified and cloned in the sense and antisense direction in either side of a *GUS* intron in plasmid vector pGAS1252 (Fig. 1). The cassette was then sub-cloned in the *Pst1* site in pCAMBIA3301, and the recombinant plasmid was designated as pCAMRNAi. The RNAi cassette was transferred into rapeseed, and putative transgenic plants were regenerated on medium containing phosphinotricin (Fig 2). Transformation efficiency of 3.7 % was obtained in SLM046 cultivar. Histochemical GUS assay in transgenic plants was done using Jefferson’s method (Jefferson et al. 1987), and blue leaf samples were observed in transgenic plants (Fig. 3). PCR analysis on T0 putative transgenic and wild-type plants showed the insertion of 501-bp antisense segment only in putative transgenic plants (Fig. 4). Seeds of putative T1 transgenic plants were sown in soil in pots, and growing plants at 5–6 leaf stage were subjected to 1.5 % basta herbicide. Three herbicide resistant lines were selected, and the insertion of 1,000-bp fragment of the *GUS* gene was confirmed by PCR (Fig. 5).

### Analysis of *BnSHP1* gene in wild-type and transgenic plants

**Fig. 6:**
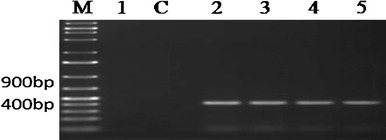
Detection of the *bar* gene in T2 transgenic *B. napus* plants by PCR. Amplification of a 400-bp fragment of the *bar* gene: *1* negative control, *c* wild type, *2–5* transgenic plants; *M* DNA marker

The effect of silencing cassette on gene expression was evaluated using T2 transgenic lines. The transgenic plants were grown in greenhouse conditions and showed normal vegetative and reproductive characteristics compared with control wild-type plants. The presence of the transgene was confirmed by amplification of a 400 bp of the *bar* gene in transgenic plants (Fig. 6).

**Fig. 7:**
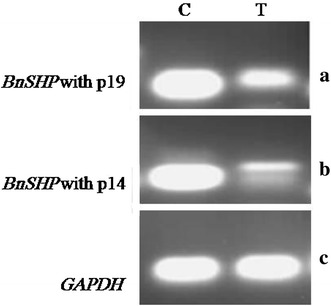
Expression analysis of *BnSHP* gene in transgenic *B. napus* plants and wild type by RT-PCR: **a** amplification of a 105-bp band using the p14 specific primers, **b** amplification of a 94-bp band using the p19 specific primers and **c** amplification of a 100-bp band using the *GAPDH* specific primers. *C* non-transgenic control plant, *T* transgenic plant

Expression of the *BnSHP* gene was analyzed using RT-PCR. A sharp 100-bp band for *BnSHP1* and *BnSHP2* gene expression was observed in wild-type plants, while transgenic plants showed a weak band as was expected (Fig. 5). The primer P14 amplifies all three *BnSHP* alleles (*SHP1, SHP2*-*a* and *SHP2*-*b*), and P19 amplifies *BnSHP1*. A very slight difference in gene expression was observed in transgenic plants. Expression level for housekeeping *GAPDH* gene as control was the same in both transgenic and control plant, indicating that expression of *BnSHP* alleles was decreased in transgenic plants as judged with the *GAPDH* gene expression (Fig. 7).

### Real-time PCR

**Fig. 8:**
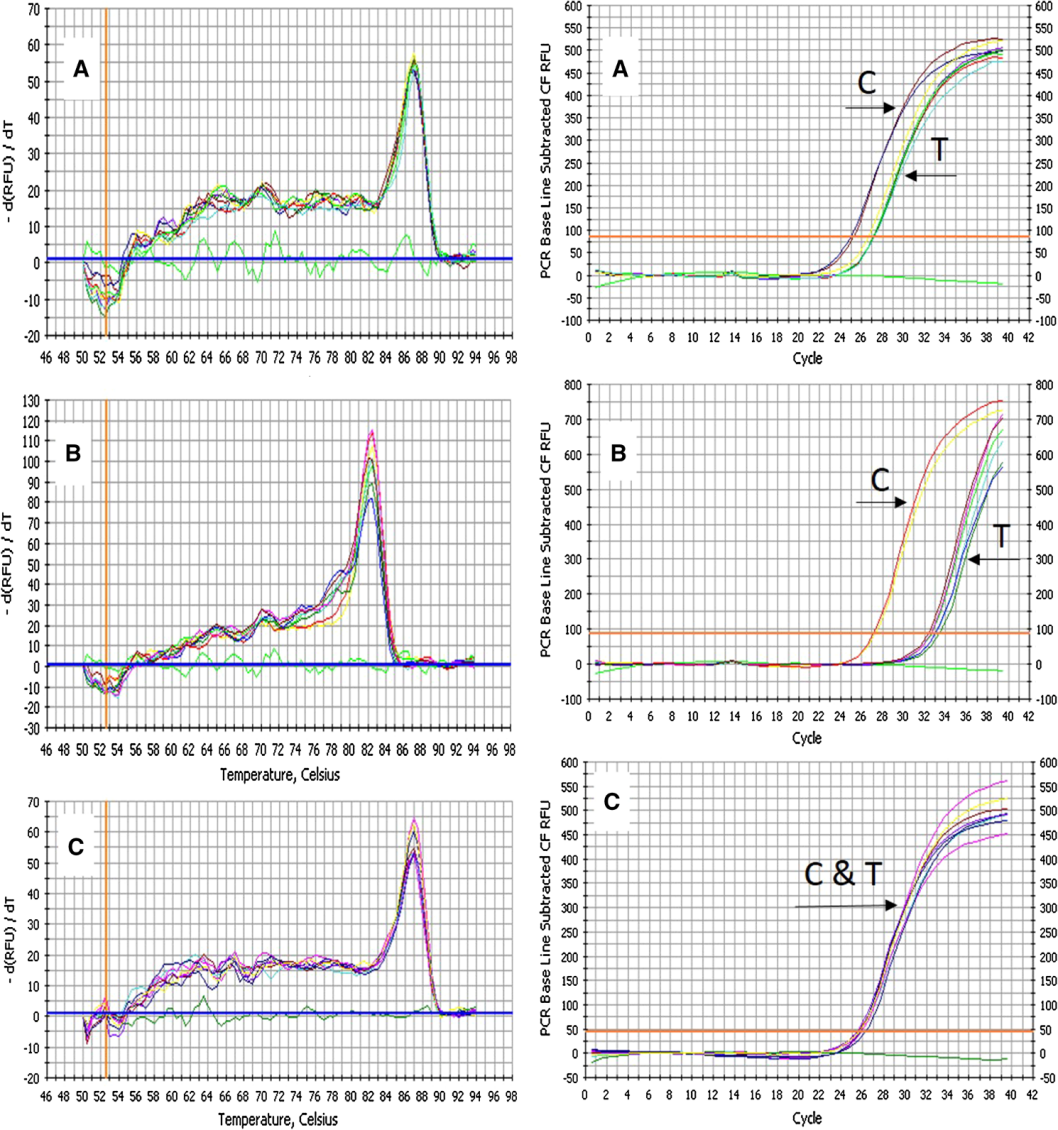
Real-time analysis of the *BnSHP*gene and *GAPDH* expression in transgenic and non-transgenic *B. napus* plant using the P19 (**a**), P14 (**b**) *BnSHP* specific primers and *GAPDH* (**c**) primers. For *BnSHP*: delay in amplification in transgenic plant indicating the reduced level of the *BnSHP*RNAs and for *GAPDH*: no difference in amplification curves, indicating the same amount of RNA in the samples. High PCR specificity is shown by melting curve analysis

**Fig. 9:**
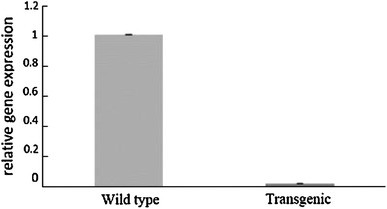
The high reduction in the *BnSHP* gene expression in *B. napus* transgenic plant compared with wild-type plant using Real-time PCR analysis

To analyze the relative changes in gene expression, a quantitative Real-time PCR using two specific primers for *BnSHP* alleles and *GAPDH* gene was applied (Fig. 8). The efficiency of amplification using dilution series was 95 and 94 % for the *GAPDH* and the *BnSHP* genes. A higher C_T_ value was observed for transgenic plants than non-transgenic plants, implying that the *BnSHP* gene expression has been decreased in transgenic plants. The data were statistically analyzed, and the related vertical graph showed the 97 % reduction in gene expression (Fig. 9).

The genetic variation in shatter resistance has been reported in Brassica species, including *B. rapa* L., *B. juncea* L., *B. hirta* L. and within wild relatives of Brassica (Kadkol et al. 1985; Wang et al. 2007, Bagheri et al. 2012). However, to avoid windrowing of crops on a routine basis, the level of resistance is insufficient (Raman et al. 2011). Random impact test (RIT) on 229 accessions of *B. napus* for shatter resistance detected only two shatter resistant lines (Wen et al. 2008). Using introgression method, shatter resistance could be improved in *B. juncea* (Kadkol 2009; Raman et al. 2011**)**. However, unwanted traits could be present too as reported by Summers et al. (2003). The DK142 line (resynthesized *B. napus* using *B. oleracea* alboglabra and *B. rapa* chinensis) showed higher shatter resistance in all locations, but significantly lower seed yield than commercial variety (Summers et al. 2003).

Indehiscent and harder siliques were obtained by constitutive MADSB expression in winter rape lines compared with wild-type winter rape plants, and precocious seed dispersal was prevented (Chandler et al. 2005). However, for unclear reason, the transgenic summer rape lines did not show a non-opening silique phenotype. This indicated that the tender rape cultivars differences could affect the co-ordination of signaling events involved in fruit dehiscence. A number of genes have been shown to involve in fruit dehiscence and seed shattering. Among the reported genes, *SHP, IND* and *ALC* genes have been suggested as ideal candidates for manipulation in breeding shatter resistant lines (Liljegren et al. 2000; Tan et al. 2009). In *B. napus*, three *SHP* alleles have been reported, and due to more probable redundancy of these alleles, resistance to shattering may need control of all these alleles simultaneously (Tan et al. 2009).

In Arabidopsis, double mutant of both *SHATTERPROOF1* (*SHP1*) and *SHATTERPROOF2* (*SHP2*) produced indehiscent pods (Liljegren et al. 2000). Pod shatter resistance was also observed in mutants of the *INDEHISCENT* gene (Liljegren et al. 2004; Wu et al. 2006), and the *ALCATRAZ* gene in Arabidopsis (Rajani and Sundaresan 2001). Sorefan et al. (2009) showed that the *INDEHISCENT (IND*) mutant produced indehiscent fruits by preventing differentiation of tissue in dehiscence zone into an abscission layer.

Over-expression of *FRUITFUL* (*FUL*), a repressor of *SHP* and *IND*, produced indehiscent siliques in Arabidopsis (Ferrandiz et al. 2000). Ectopic expression of *FUL* gene in *B. juncea* produced indehiscent fruits, but they could not be threshed in a combine harvester without seed damage (Ostergaard et al. 2006). In the present study, introduction of the *BnSHP* gene-silencing cassette into *B. napus* showed a drastic reduction in *BnSHP* expression. Morphologically, transgenic plants were normal and set seeds at greenhouse conditions. At ripening stage, pods failed to shatter. Further phenotypic evaluation of shatter resistance such as the cantilever test, pendulum test (Kadkol et al. 1984) and the RIT are needed to be done in produced transgenic plants.
